# Characterization of Human Cytomegalovirus (HCMV) Long Non-Coding RNA1.2 During Lytic Replication

**DOI:** 10.3390/v17020149

**Published:** 2025-01-23

**Authors:** Salomé Manska, Andrew Hagemann, Janna Magana, Cyprian C. Rossetto, Subhash C. Verma

**Affiliations:** Department of Microbiology and Immunology, University of Nevada, Reno School of Medicine, Reno, NV 89557, USA; salomemanska@gmail.com (S.M.); andrewhagemann62@gmail.com (A.H.); jmagana@med.unr.edu (J.M.); crossetto@med.unr.edu (C.C.R.)

**Keywords:** HCMV, lncRNA, lytic replication

## Abstract

During lytic replication of human cytomegalovirus (HCMV), the most abundant viral transcripts are long non-coding RNAs (lncRNAs). Viral lncRNAs can have a variety of functions, some of which are necessary for viral production and the modulation of host processes during infection. HCMV produces four lncRNAs, Beta2.7 (RNA2.7), RNA4.9, RNA5.0 and RNA1.2. While there has been research on these viral lncRNAs, many of their functions remain uncharacterized. To determine the function of RNA1.2, we explored its requirement during lytic infection by generating viral mutants containing either a full or partial deletion of the RNA1.2 locus. Within permissive fibroblasts, the RNA1.2 deletion mutants showed no defects in viral DNA synthesis, transcript expression, protein production, or generation of viral progeny. Further investigation to identify potential cellular and viral protein binding partners of RNA1.2 was performed using liquid chromatography-mass spectrometry (LC-MS). A significant number of cellular proteins were identified and associated with RNA1.2. Specifically those associated with the innate immune response, mitochondrial processes, and cell cycle regulation. While RNA1.2 is dispensable for lytic replication, these findings suggest it may play a pivotal role in modulating the host response.

## 1. Introduction

Human cytomegalovirus (HCMV) infections are ubiquitous worldwide. It is estimated that the seroprevalence of HCMV ranges from 45–100%, with low- and middle-income communities experiencing the highest rates of infection [1]. While healthy individuals experience primarily asymptomatic infection, HCMV causes severe disease in immunocompromised and at-risk populations. Some examples of those who experience severe or life-threatening complications are transplant recipients, newborn infants, and HIV/AIDS patients [2]. Currently, there is no approved vaccine for HCMV, and long-term use of some HCMV antivirals such as ganciclovir (GCV), valganciclovir (VGV), maribavir (MBV) or phosphonomethanoic acid (foscarnet), can lead to resistance or potential toxicity with continual use [3,4,5].

As a member of the herpesviridae family, HCMV establishes a lifelong infection in human hosts following initial infection. The primary site of latency for HCMV occurs in bone marrow cells of the myeloid lineage [6]. During this phase, HCMV DNA persists without producing progeny virions, although specific viral transcripts remain detectable [7]. Upon reactivation, the differentiation of myeloid progenitor cells triggers lytic replication, producing new viral progeny. During the productive lytic phase, viral gene expression occurs in a precise temporal cascade, encompassing immediate-early (IE), early (E), and late (L) genes.

HCMV has the largest genome amongst all known human herpesviruses. The linear dsDNA genome consists of ~236 kb and has approximately 180 genes [8]. Most of these genes are protein-coding, but there are four highly abundant non-coding transcripts [9]. Previous transcriptome analysis during a lytic HCMV infection identified that 65.1% of viral polyadenylated transcripts are long non-coding RNAs (lncRNAs). lncRNAs are characterized as >200 nucleotides (nts) in length and have no apparent protein-coding potential [10]. The four identified HCMV lncRNas are RNA1.2, RNA2.7, RNA4.9, and RNA5.0, which are named according to the approximate length of their RNA molecules (kb) [11]. The expression of the lncRNAs at high levels, requiring finite resources, suggests that these transcripts play a role and are major regulators in viral replication and host biological processes during infection [12].

Investigation of the functional roles of viral lncRNAs has expanded in recent years. Once hypothesized as “dark matter” or “junk” nucleic acid, these lncRNAs have proven to play an integral part in biological processes [10]. The functions of lncRNAs can vary widely, but in general, they can serve as guides to recruit proteins to specific DNA sequences, act as decoys to titrate proteins within specific areas of the cell, form scaffolds to assemble multi-protein complexes, and interact directly with RNA or DNA to regulate translation or RNA processing. ncRNAs have been detected in many different viruses, including other herpesviruses such as Kaposi’s sarcoma-associated herpesvirus (KSHV) Polyadenylated Nuclear transcript (PAN) RNA, and herpes simplex virus 1 (HSV-1) Latency associated transcript (LAT), and have functional capabilities that can increase viral replication efficiency and modulate innate antiviral response mechanisms [13].

Of the four HCMV lncRNAs, RNA2.7 is the most abundant at over 46.8% of all viral polyadenylated transcripts [11]. RNA2.7 is an early transcript (also referred to as Beta2.7 because of its expression kinetics) from promoter response to immediate-early protein 1 and 2 (IE1 and IE2) [14,15]. There is also evidence that RNA2.7 is expressed during latency [16,17]. RNA2.7 modulates cell cycle progression and apoptosis by interacting with host factors such as DNA replication factor 1 (Cdt1), cell division cycle gene 6 (Cdc6), and the mitochondrial complex I [18]. The interaction between RNA2.7 and the GRIM-19 subunit of mitochondrial complex I has been shown to protect infected cells from apoptosis by maintaining ATP production from the mitochondria [18]. This has led to studies on the therapeutic potential of RNA2.7 in the absence of infection for ischemia and Parkinson’s disease [19,20]. Recent studies have also pointed to RNA2.7 function in upregulating cellular gene expression involved in cell motility and facilitating virus cell-to-cell spread [21].

Approximately 10.1% of the viral polyadenylated transcripts are RNA4.9 [11]. RNA4.9 is localized to the host nuclear compartment and may be involved in initiating viral origin replication [22]. HCMV viral DNA replication begins at a single origin, the origin of lytic DNA replication (*ori*Lyt). After RNA4.9 is transcribed, it forms an R-loop and becomes embedded within the *ori*Lyt region, creating an RNA–DNA hybrid structure [22,23]. The RNA stem–loop structure, formed at the 5′ end of RNA4.9, is recognized by the proposed origin binding protein (OBP) UL84 for initiation of viral DNA replication [24]. Disruption of the RNA4.9 promoter leads to a decrease in the single-stranded DNA binding protein (ssDBP) UL57, the transcription of which originates upstream from *ori*Lyt [21]. RNA4.9 has also been proposed to play a role during latency to facilitate the repression of the major immediate-early promoter (MIEP) by recruiting the polycomb repression complex for chromatin remodeling [16].

Among the HCMV viral lncRNAs, RNA5.0 and RNA1.2 remain the least characterized. RNA5.0 is a stable intron and was shown to be dispensable for replication in fibroblasts but may play a role in viral pathogenesis; in a murine model of infection with MCMV, the homologue of RNA5.0 (RNA7.2) was shown to be required for viral persistence [25,26,27]. The differences in the results between HCMV RNA5.0 in a cell culture model and MCMV RNA7.2 in the murine infection model highlight the limitations of in vitro systems, which cannot recapitulate the infection dynamics of a whole organism.

RNA1.2 accounts for 7.9% of the polyadenylated viral transcripts [11]. The characterization of RNA1.2 remains limited, but studies in HCMV strain Merlin identified that it modulates host innate immune responses by blocking NF-κB activation and preventing the release of IL-6 [12]. This study generated and characterized two different RNA1.2 viral mutants using the Merlin bacterial artificial chromosome (BAC) system, one lacking most of the RNA1.2 gene locus and the other a deletion of only the TATA sequence within the promoter. In a fibroblast infection model, neither RNA1.2 mutants had significant viral growth defects and showed minimal differences in viral gene expression compared to wild-type viruses. Interestingly, differential expression analysis identified significant regulation differences in 76 cellular genes, both at the RNA and protein level, in the RNA1.2 mutant viruses. The most highly upregulated was Tumor Protein p63-Regulated Gene 1-Like protein (TPRG1L), one of the known functions of which is to increase the expression of IL-6 through NF-κB activation. An increase in the expression of chemokines MCP-1 and CXCL1 in the mutants lacking RNA1.2 was further demonstrated.

A recent report explored the expression and function of HCMV lncRNA during latency and lytic reactivation using strain Toledo in Kasumi-3 and fibroblast infection models [28]. Using nanopore direct RNA sequencing (DRS), selecting for polyadenylated transcripts, the authors found that RNA1.2 accounted for ~17% of poly-A viral transcripts during latency, reactivation, and lytic infection. Interestingly, using an RNA1.2 deletion virus in the Toledo BAC, they reported a significant decrease in cell-free virus particles in the supernatant compared to WT in permissive fibroblasts [28]. Using the same virus in a Kasumi-3 model of infection, they suggest RNA1.2 may play a role in maintaining the latent viral DNA reservoir and is required for productive reactivation. They assessed the epitranscriptome, finding no splicing junctions within RNA1.2, and noted that differential RNA modification sites between latency and other infection states (reactivation and lytic) were more frequent in viral lncRNAs, including RNA1.2, which indicates post-transcriptional regulation through RNA modifications. Finally, they used RNA antisense purification coupled with mass spectrometry (RAP-MS) to identify proteins interacting with RNA1.2, which included viral proteins IRS1, UL35, UL47, cellular proteins associated with the ER-Golgi network (MYO1B, MYO6, and SEC23IP), proteins involved in antiviral response, and cellular m6A readers IGF2BP3 and YTHDF2.

The prior studies characterizing RNA1.2 utilized the HCMV strains Merlin and Toledo. Notably, RNA1.2 has been reported to exhibit minimal variability between strains, underscoring its conserved nature across different viral lineages [29,30]. However, there are some differences in the conclusions regarding the requirement of RNA1.2 for productive replication [12,28]. To better understand the potential role of RNA1.2, we generated RNA1.2 mutants in HCMV strain TB40E and assessed viral DNA synthesis, RNA and protein expression, and production of infectious virions. In our studies, RNA1.2 was determined to be dispensable for lytic replication in permissive human fibroblasts. To further address the potential functional role of RNA1.2, we used proteomics to identify cellular and viral proteins that interact with RNA1.2. For these experiments, RNA1.2 was in vitro transcribed using biotinylated UTP and the labeled RNA was incubated with either infected or non-infected cell lysate, followed by LC-MS to identify protein interactions. We report numerous viral and cellular proteins associated with RNA1.2, and there interactions suggest that its function may be to modify the host cell environment rather than directly participating in viral replication.

## 2. Materials and Methods

### 2.1. Cells and Viruses

Human fibroblast (HF) cells were cultured in Dulbecco’s modified Eagle medium (DMEM) supplemented with 10% fetal bovine serum (Corning) [31]. Cells were maintained at 37 °C in a 5% CO_2_ environment. The TB40E WT, TB40E Δ1.2, and TB40E Δ1.2 half viral stocks were grown and titrated on HF cells, and stocks were aliquoted and stored at −80 °C.

### 2.2. Generating the TB40E Δ1.2 Half and TB40E Δ1.2 Viral Mutants

TB40E WT BACmid DNA was used to generate the viral mutants containing deletions of the RNA1.2 genetic locus. Half and complete deletions of RNA1.2 were removed by BAC recombineering methods via homologous recombination. For Δ1.2 half mutants, gBlocks (IDT) were designed to contain a kanamycin cassette with homologous arms that flank the middle of the RNA1.2 gene and the 3′ end to generate the Δ1.2 half mutant (Table 1). For the Δ1.2 mutant, the gBlock contained a kanamycin cassette with homologous arms flanking regions that overlap the 5′ and 3′ end of the RNA1.2, resulting in a complete gene deletion (Table 1).

The homologous recombination step to replace the RNA1.2 gene sequence with kanamycin was performed in *E. coli* (GS1783) that harbored TB40E WT BAC DNA. To generate GS1783 competent cells containing the viral BAC DNA, bacteria were grown in LB with 30 μg/mL of chloramphenicol with shaking at 32 °C. From the overnight culture, a fraction of the grown cells was cultured in 12.5 mL LB with chloramphenicol and grown at 32 °C with shaking until OD_600_ reached 0.5–0.6. The bacterial cells were then immediately incubated in a 42 °C water bath for 15 min to induce the expression of the recombination enzymes. The cells were then cooled in an ice bath and pelleted at 4000 rpm at 4 °C for 10 min. The bacteria pellet was washed with H_2_O and pelleted. After this first wash, a second wash was performed. The pellet was then resuspended in 1 mL of H_2_O and centrifuged at 13,000 rpm for 20–30 s at 4 °C. These washes were repeated twice with a final resuspension in 100 μL of water for immediate use. Importantly, all washes were performed with ice-cold H_2_O. To reserve competent bacteria, the cells were resuspended in 10% glycerol, followed by flash freezing in an ethanol dry-ice bath and storing cells at −80 °C.

To perform homologous recombination, 30 μL of competent cells were electroporated with 1 μL of gBlock DNA (10–30 ng DNA) using the Cell-Porator (Gibco BRL) set at 330 uF capacitance and 400 volts with a 2 mm cuvette. Bacteria was recovered in 1 mL of warmed LB and incubated for 1.5 h at 32 °C while shaking. The cells were then centrifuged at 4000 rpm for 4 min, followed by removing supernatant. Cells were then gently resuspended in residual LB and plated onto LB-agar plates containing chloramphenicol (30 μg/mL) and kanamycin (50 μg/mL). Plates were incubated overnight at 32 °C. Bacterial colonies were grown as liquid cultures in LB with chloramphenicol and kanamycin overnight. Bacteria were pelleted, and DNA was isolated using alkaline lysis and phenol-chloroform extraction.

The Kan cassette was removed by growing bacteria harboring mutant BAC DNA in the presence of arabinose to induce the Isce enzyme. First, 0.5 mL of mutant BAC DNA containing the kanamycin cassette was grown overnight in 10 mL LB and chloramphenicol. A 2 mL fraction of the culture was then grown in 2 mL of LB with 2% L-arabinose for a final medium concentration of 1% L-arabinose. The cultures were then incubated at 32 °C for 1 h followed by 42 °C for 20 min to induce expression of the recombination enzymes. The cultures were returned to a 32 °C shaking incubator for 3–3.5 h. From here, cultures were diluted and spread onto LB-agar plates containing 1% arabinose and chloramphenicol and grown overnight at 32 °C. Bacterial colonies were picked and grown in LB with chloramphenicol. BAC DNA was purified from these cultures using the NucleoBond Xtra BAC kit (Clontech) per the manufacturer’s protocol.

Whole genome sequencing of BAC DNA from TB40E WT, TB40E Δ1.2, and TB40E Δ1.2 half was performed by the Nevada Genomics Center using the Illumina NextSeq500. Fastq files were aligned to an HCMV reference genome (EF999921-TB40E) in CLC Genomics Workbench (Qiagen). Sequencing data were deposited to the NCBI Sequence Read Archive (SRA) and can be retrieved with the following SRA accessions: TB40E WT, SRR14136388; TB40E Δ1.2, SRR14136387; and TB40E Δ1.2 half, SRR14136386.

To generate a virus from the purified viral BAC DNA, HF cells were transfected with BAC DNA (2–3 μL) and a plasmid containing pp65 (0.5 μg) using the Human Dermal Fibroblast Nucleofector Kit (Lonza, #VPD-1001) as described previously. Transfected cells were maintained until viral production began and subsequent spread was detected. The infected cells were harvested, and the virus produced was purified by freeze-thawing and pelleting. The virus was titrated for further experimentation.

### 2.3. Immunofluorescent Imaging

HF cells were plated onto coverslips and grown to super-confluency. Cells were infected with TB40E WT, TB40E Δ1.2, and TB40E Δ1.2 half (MOI = 1). Media were removed, and cells were washed with PBS, followed by fixation with 4% paraformaldehyde at room temperature for 15 min. All subsequent washes were performed using 3% BSA in PBS. Cells were permeabilized with 0.5% Triton X-100 in PBS for 20 min at room temperature. Immunostaining was performed after the permeabilization step. Cells were blocked with 3% goat serum in PBS for one hour at room temperature, followed by antibody labeling. Primary antibodies used were UL44 1:500 (Fitzgerald, Acton, USA, 10-C50I), UL84 1:1000 [32], IE2 1:1000 (Millipore, Burlington, USA, MAB8140), and UL57 1:500 (Virusys, Milford, USA, P1209). Primary antibodies were incubated on cells overnight at 4 °C. The next day, cells were washed and incubated with fluorescent antibodies: Alexa Fluor 594 anti-mouse 1:2000 (Invitrogen, Carlsbad, USA, #A21201) and Alexa Fluor 488 anti-mouse 1:2000 (Invitrogen, #A11029). Coverslips were mounted onto glass slides using ProLong Gold Antifade reagent with DAPI (Invitrogen). Slides were imaged using a fluorescent microscope (Carl Zeiss Inc., White Plains, USA, Inc.).

### 2.4. Growth Curve

HF cells were first seeded onto 12-well plates. After cells were confluent (2–3 days post-plating), the cells were infected with TB40E WT, TB40E Δ1.2, and TB40E Δ1.2 half at MOI = 1.0 or MOI = 0.1. Cell-free virus in the lysate (1 mL) was collected from a well every 3 days after infection: 3 dpi, 6 dpi, 9 dpi, and 12 dpi. Collected samples were centrifuged at 13,000 RPM for 5 min at 4 °C to pellet cellular debris. Clarified infectious lysate was then used to perform 10-fold serial dilutions of each collected sample. The serially diluted lysate was then used to infect additional confluent HF cells in 12-well plates. The cells were incubated with infectious lysate for two hours for viral adsorption. The media were then removed, and cells were overlaid with 0.4% agarose in DMEM media. After 5–7 days, GFP plaque formations were counted and plotted.

### 2.5. qPCR Analysis

HF cells were infected with TB40E WT, TB40E Δ1.2, and TB40E Δ1.2 half (MOI = 1). After 4 hpi, 24 hpi, 48 hpi, and 72 hpi, cell lysate was harvested, and RNA was isolated using the NucleoSpin Plasmid EasyPure Kit (Macherey-Nagel, Duren, Germany, #740727.250). The RNA was then treated with DNase using the TURBO DNA-free Kit (Invitrogen, #AM1907) followed by adding 1 µL RNaseOUT (Invitrogen, #10777-019). After RNA was purified, a cDNA synthesis reaction was performed using the iScript cDNA Synthesis Kit (Bio-Rad USA, #1708891). cDNA was then quantified by qPCR analysis using primers and probes specific to RNA1.2, RNA4.9, RNA B2.7, IE2, UL54, and 7sk (Table 2).

### 2.6. Immunoblotting

HF cells were infected with TB40E WT, TB40E Δ1.2, and TB40E Δ1.2 half (MOI = 1). After 24 hpi and 72 hpi, cells were washed with 1X PBS and lysed using RIPA buffer (Thermo Scientific USA, 89901) with protease inhibitor cocktail (Sigma USA, P8340). Cells were scraped and harvested. Samples were then sheared using a probe sonicator at 30 amplitudes for a total of 30 s: 10 s on and 10 s off. Cellular debris was then centrifuged at 13,000 RPM for 10 min at 4 °C. Protein was denatured using Laemmli Buffer (Bio-Rad) with β-mercaptoethanol and boiled at 95 °C for 5 min. The protein was then resolved by SDS-PAGE and transferred to a PVDF membrane (Bio-Rad) for immunoblotting analysis. The membrane was blocked in 5% BSA in Tris-buffered saline, 0.05% Tween-20 (1X TBST) for 30 min. The membrane containing samples from 24 hpi was then probed with antibodies to detect IE1/IE2 1:1000 (Virusys, P1215) and H3 1:5000 (Abcam USA, ab1791). Membrane with samples from 72 hpi was probed with antibodies to detect UL44 1:1000 (Fitzgerald, 10-C50I), pp65 1:150 (Abcam, ab49214), and H3. Protein was detected by incubation with secondary antibody using IRDye 680 anti-mouse Ab 1:10,000 (LI-COR USA, #926-68070) and IRDye 800 anti-rabbit Ab 1:10,000 (LI-COR, #926-32211).

### 2.7. Biotinylated RNA1.2 In Vitro Transcription

The genetic locus of RNA1.2 was synthesized from a g-Block (Table 3). Homologous recombination was performed to clone the RNA1.2 locus into the pGEM vector using the GeneArt Seamless Cloning and Assembly (cat. A13288). Constructs were transformed using One Shot TOP10 chemically competent *E. coli* (cat. C404010) according to the manufacturer’s protocol. The pGEM-RNA1.2 construct was tested by restriction digest and verified by Sanger sequencing.

To generate the pGEM-RNA1.2 plasmid construct, pGEM 7zf- was linearized using the HindIII restriction enzyme. RNA1.2 was then in vitro transcribed from the pGEM-RNA1.2 plasmid construct using the MEGAscript T7 Kit (Thermo Fisher, #AM1334). Generation of biotinylated RNA was performed using the Biotin RNA Labeling Mix (Sigma-Aldrich, #11685597910) in substitution of non-biotinylated nucleotides in the kit. As a control, non-biotinylated RNA 1.2 was also synthesized. As recommended by the manufacturer, transcription reactions proceeded for four hours at 37 °C. RNA was then treated with DNase as described above, followed by adding 1 μL of RNaseOUT.

A LiCl precipitation was used to precipitate RNA from solution and to remove unincorporated nucleotides. Precipitated RNA was then resuspended in 100 µL nuclease-free water, solubilized in a 65 °C water bath for 15 min, and checked for concentration and contamination by Nanodrop. RNA was resolved on a 1.25% agarose-formaldehyde/MOPS gel for two hours at 65 volts to assess length and integrity.

Following RNA gel analysis, a biotinylated northern blot was performed. RNA was transferred overnight to a Zetaprobe membrane using 2X SSC as the transfer buffer. After transfer, RNA was UV-crosslinked to the membrane for one minute. After crosslinking, the membrane was transferred to a LI-COR black box and blocked with Odyssey blocking buffer (LI-COR, #927-60001) and 1% SDS (Thermo Fisher, #BP1311-1), with rocking at room temperature for 30 min. The blocking buffer was removed, and a 1:2500 dilution of IRDye 800CW Streptavidin (LI-COR, #926-32230) in Odyssey blocking buffer + 1% SDS was added to the membrane. Streptavidin binding occurred by rocking at room temperature for 30 min. After streptavidin incubation, the streptavidin solution was removed. The blot was washed thrice with 1X TBST for 5 min each, rocking at room temperature. A final rinse was done in 1X PBS. The blot was imaged on the BIO-RAD ChemiDoc MP Imaging System.

### 2.8. Isolating RNA-Protein Complexes

Following a 72-h infection of HFs with TB40E in 10 cm dishes, cells were scraped and resuspended in 500 µL of NP-40 lysis buffer (50 mM Tris-HCl, 150 mM NaCl, 5 mM EDTA, 1% NP-40) + protease inhibitor. Cells were sonicated 2X for 10 s. Cellular debris were centrifuged, and the supernatant was transferred to a new tube with 1100 µL NP-40 lysis buffer, leading to a total of 1600 µL. A quantity of 10 µL cell lysate was saved for the input, and the remaining lysate was aliquoted into two tubes. A quantity of 25 µL of Dynabeads MyOne Streptavidin T1 magnetic beads (Thermo Fisher, #65601) for each sample was washed with one milliliter of NP-40 lysis buffer and resuspended in 25 mL of NP-40 lysis buffer. Washed magnetic beads were added to the cell lysate and rotated at room temperature for 20 min to preclear the lysate. After incubation, beads were discarded.

A quantity of 10 µg of non-biotinylated or biotinylated RNA1.2 and 5 µL of RNaseOUT were added to 800 µL of precleared cell lysate, either mock or TB40E-infected cells, and incubated for one hour, rotating at room temperature. A quantity of 25 µL of washed T1 magnetic beads was then added to each sample and rotated for 30 min at room temperature. Each tube was then placed on a magnetic stand to separate the beads from the lysate. Lysate was discarded and the beads were washed 3X with one milliliter of TBS + 1% Triton X-100 for 10 min, rotating at room temperature. Following the final wash, the beads were resuspended in 50 µL of Laemmli Sample Buffer (Bio-Rad, #161-0737) + β-mercaptoethanol (β-ME). A quantity of 40 µL of Laemmli + β-ME was added to the input sample. All samples were boiled for five minutes and run on SDS-PAGE gel followed by silver staining using the Pierce Silver Stain Kit (Thermofisher, #24612) or Coomassie staining for proteomic analysis.

### 2.9. Mass Spectrometry and Data Analysis

Proteins isolated from the RNA1.2 pulldown were resolved on an AnykD Mini-Protean TGX protein gel (bio-rad) for 6 min at 165 volts. Once the sample was in the gel, proteins were stained with Coomassie Brilliant Blue, and the blue portion of the gel was excised for analysis. Samples were submitted to the Mick Hitchcock Ph.D., Nevada Proteomics Center at the University of Nevada, Reno, where LC-MS was performed. After LC-MS, Scaffold 4.10.0 was used to sort peptide counts and set stringency conditions. The proteomic data were analyzed with a peptide false discovery rate (FDR) of 0.9%, protein FDR 0.0%, peptide threshold 95% minimum, and protein threshold 99% minimum. For the pie charts and heat maps, proteins were identified as significant if peptide count was greater than three-fold compared to non-biotinylated RNA controls. Raw data can be found in Supplemental Table S1.

### 2.10. RNA-Immunoprecipitation (RNA-IP)

To perform the RNA-IPs, a previously described protocol was modified [33]. HF cells were grown to confluence in six 10 cm tissue culture dishes. Half of the cells were non-infected or infected with TB40E WT (MOI = 1). After 72 hpi, cells were fixed using 1% formaldehyde for 15 min RT and quenched with 250 mM glycine for 5 min RT. Cells were washed with PBS and harvested in 250 µL of Pierce IP lysis buffer (Thermo Fisher, #87788), RNaseOUT, and 1mM PMSF. Three dishes of either non-infected or infected cell lysate were pooled together for a total volume of ~750 mL. The lysate was then sonicated using a probe sonicator under the following conditions: 35 amp, 10 pulses, 20 s On, 30 s Off. Cell debris were pelleted by centrifuging at 800× *g* for 5 min at 4 °C. An amount of 10% of the total lysate was saved for input.

Before IP reactions were prepared, Protein G beads (Invitrogen, #10004D) were blocked in 2% BSA in PBS and with 10 µg/mL of yeast RNA (ambion, #AM7118) for 30 min rotating at RT. To precipitate RNA–Protein complexes, IP reactions contained 30 µL of pre-blocked Protein G Beads, and 1.5 µg of the following antibodies: RecQ1 (Santa Cruz, USA, #H-110), UL44 (Fitzgerald, 10-C50I), H2A (abcam, #ab18225), or 1 µg IgG (Diagenode, Denville, USA, C15410206). Clarified lysate and RNaseOUT were added to the IP reaction and incubated overnight at 4 °C while rotating.

The next day, samples were washed once with the following buffers, a low-salt buffer (0.1% SDS, 1% Triton X-100, 2 mM EDTA, 20 mM Tris-HCl pH 7.0, and 150 mM NaCl), high-salt buffer (0.1% SDS, 1% Triton X-100, 2 mM EDTA, 20 mM Tris-HCl pH 7, and 500 mM NaCl), LiCl Wash (0.25 M LiCl, 1% NP-40, 1% Na-deoxycholate, 1 mM EDTA, and 10 mM Tris-HCl pH 7.0), and two final washes in 1X TE. Samples were decrosslinked and RNA was released by resuspending beads in 200 µL of Reverse buffer (10 mM Tris-HCl pH 7.0, 5 mM EDTA, 10 mM DTT, and 1% SDS) and adding 100 µL of reverse buffer to input. All samples were incubated with the addition of 200 µL of proteinase K solution (10 mM Tris-HCl pH 7.0, 1mM EDTA, 0.5% SDS, 100 mM NaCl, and 5% proteinase K). The input and beads were incubated for 1 h at 42 °C followed by 2 h at 65 °C while rotating.

RNA was extracted using Trizol LS (Ambion, USA, #10296010) according to the manufacturer’s protocol. Samples were then treated with DNase and with RNaseOUT as previously described. The RNA was then used for cDNA synthesis and qPCR analyses as described above. To detect cDNA from isolated transcripts, primers and probes specific to RNA1.2 and GAPDH were used.

### 2.11. Statistical Analysis

Statistical analyses were completed using one-way ANOVA with Tukey’s multiple comparison tests, two-way ANOVA with Bonferroni’s multiple comparison test, or Student’s *t*-test with multiple comparisons. All statistical analyses were completed using GraphPad Prism software version 9.0.1.

## 3. Results

### 3.1. Construction and Characterization of RNA1.2 Mutants

**Figure 1:**
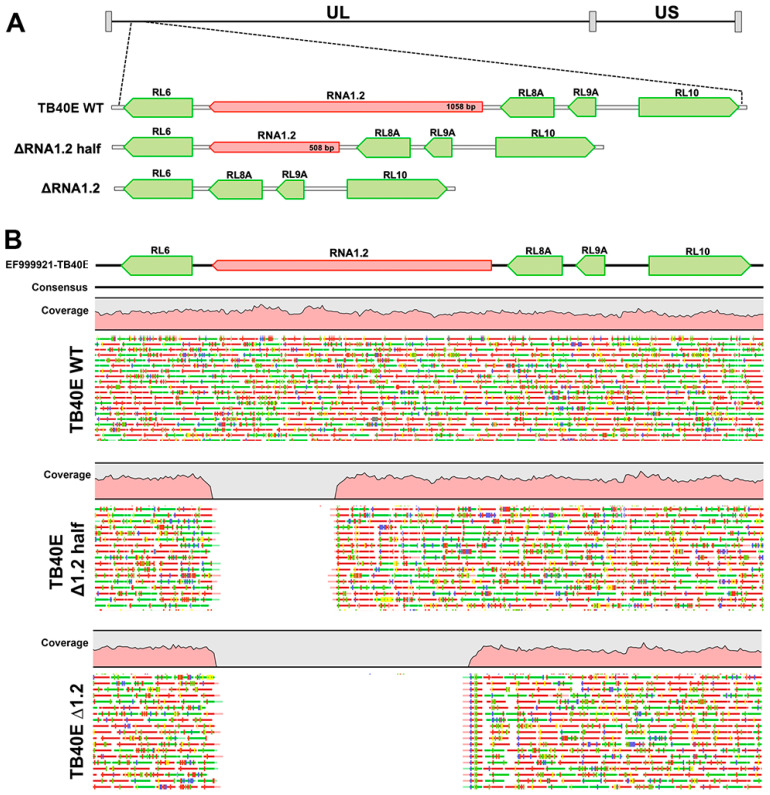
Whole genome sequencing analysis of TB40E ΔRNA1.2 half and TB40E ΔRNA1.2 mutants. TB40E mutants with partial and complete deletions of RNA1.2 were generated using BAC recombineering. (**A**) A schematic of the genetic locus indicating the deletions of RNA 1.2 in the HCMV genome, RNA1.2 genetic locus is in red and surrounding genes are shown in green. (**B**) WGS was performed to confirm half and complete deletions of RNA1.2 compared to TB40E WT. Qiagen CLC genomics workbench was used to map sequencing reads to a reference HCMV genome.

To determine if RNA1.2 is required for lytic replication, RNA1.2 mutants were generated in HCMV strain TB40E by bacterial artificial chromosome (BAC) recombineering methods. Two different mutants were constructed to disrupt RNA1.2, one containing a complete removal of RNA1.2 (ΔRNA1.2) while the other was a half deletion from approximately the middle to the 3′ end of the gene (ΔRNA1.2 half) (Figure 1A). The seamless removal of either the full or partial RNA1.2 locus was generated via homologous recombination and confirmed by whole genome sequencing and alignment to the wild-type (WT) genome (TB40E) (Figure 1B). No other off-target mutations or deletions were found when aligned to the WT DNA sequence. Stocks of WT, ΔRNA1.2, and ΔRNA1.2 half virus were made by transfecting the BAC DNA into permissive human fibroblasts (HF) along with a pp71 expression plasmid. The initial transfected cells and lysate were collected and used to seed larger flasks of HF cells to propagate the viruses further. Viral stocks used for the experiments were purified by centrifugation, and viral titers were calculated from plaque assays of stock serial dilutions on HF cells. Note that while we performed whole genome sequencing on the viral BAC DNA to confirm the targeted mutations of RNA1.2 and no other genomic changes, we did not perform whole genome sequencing on the viral stocks after transfection and passage in fibroblasts. To mitigate potential lab-adapted mutations, we limited the number of passages, but there could have been unrecognized genomic changes that were acquired during the process of transfection and passage in fibroblasts.

A mutant that only contained a partial removal of RNA1.2 was created for two reasons. First, we assumed that if the whole deletion resulted in an altered phenotype, a half deletion would aid in determining if there is a specific functional domain within the 3′ or 5′ region. Additionally, we were interested in keeping the 5′ region intact because potential splice donor or acceptor sites for leftward transcripts and small ORFs have been reported within the 5′ region of RNA1.2 [8,11]. Ribosomal profiling with mass spectrometry has suggested potential small polycistronic ORFs within the 5′ region of RNA1.2, although these potential ORFs were noted to have low translation efficiency [8]. For these reasons, the subsequent experiments performed in this study used both ΔRNA1.2 and ΔRNA1.2 half.

### 3.2. Deletion of RNA1.2 Does Not Disrupt Viral Gene Expression or Protein Production

**Figure 2:**
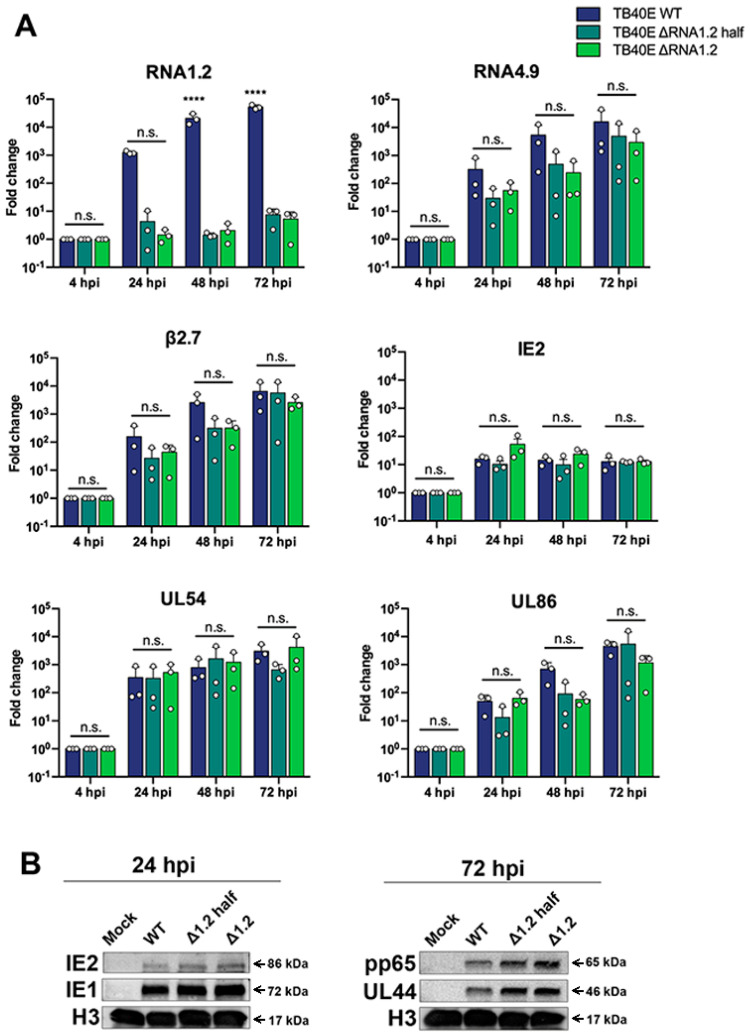
Viral gene expression and protein analysis TB40E WT, TB40E ΔRNA1.2 half, and TB40E ΔRNA1.2. (**A**) HF cells were infected with TB40E WT, ΔRNA1.2 half, and ΔRNA1.2 (MOI = 1). Cell lysate was harvested at 4 hpi, 24 hpi, 48 hpi, and 72 hpi. RNA was isolated, and transcript expression was measured by qPCR analysis using primers and probes specific to RNA1.2, RNA4.9, RNA B2.7, IE2, UL54, UL86 and 7SK (cellular). Fold change of each transcript was calculated using ΔΔCT, normalized with cellular 7SK and compared to the 4 hpi time point. Error bars indicate S.D. from independent experiments (N = 3). Statistical analysis was performed using two-way ANOVA, ****, *t*-test; n.s., not significant. (**B**) For protein analysis, cells were infected as described above. The mock and infected cell lysate was harvested at 24 hpi and 72 hpi. Cell lysate was denatured, and proteins were separated by SDS PAGE and transferred to PVDF membrane for Western blot. Proteins were identified using antibodies specific for labeling IE2, IE1, pp65, UL44, and H3.

To characterize the RNA1.2 mutants, we began by assessing viral transcript expression during lytic replication. Following infection, total RNA was harvested at 4 hpi, 24 hpi, 48 hpi, and 72 hpi, treated with DNAse and used to generate cDNA for qPCR. Fold enrichment of viral transcripts was calculated in the RNA1.2 mutants compared to WT during infection (Figure 2A). Representative temporal classes of transcripts were measured; this included IE2 (immediate-early), UL54 (early), and UL86 (late). These viral transcripts and other viral lncRNAs tested, RNA4.9 and β2.7, showed no significant differences in transcript production. RNA1.2 expression was the only transcript that showed a significant difference between WT and the RNA1.2 mutants.

We next investigated viral protein expression in TB40E WT, ΔRNA1.2 half, and ΔRNA1.2. Mock and infected cell lysate were harvested after 24 hpi and 72 hpi and analyzed by Western blot (Figure 2B). At 24 hpi we assessed the immediate early proteins IE1 and IE2 and found comparable levels between WT and the ΔRNA1.2 mutants. For the 72 hpi samples, the viral processivity factor UL44 (early) and tegument pp65 (late) proteins also showed similar expression between WT and the RNA1.2 mutants. Western blots were re-probed for Histone H3 as a loading control and showed comparable levels of proteins across all samples.

**Figure 3:**
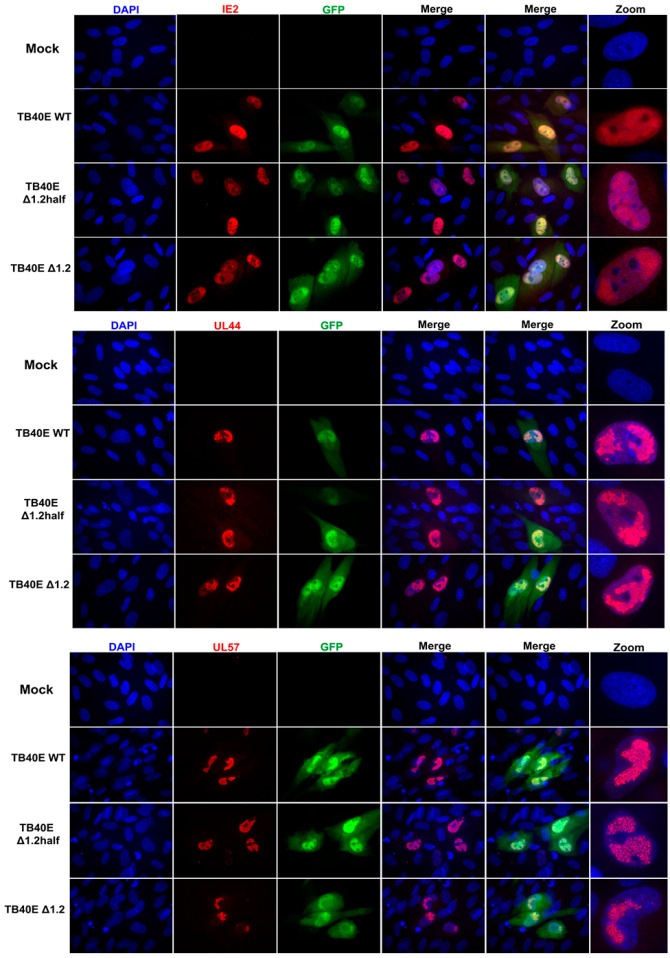

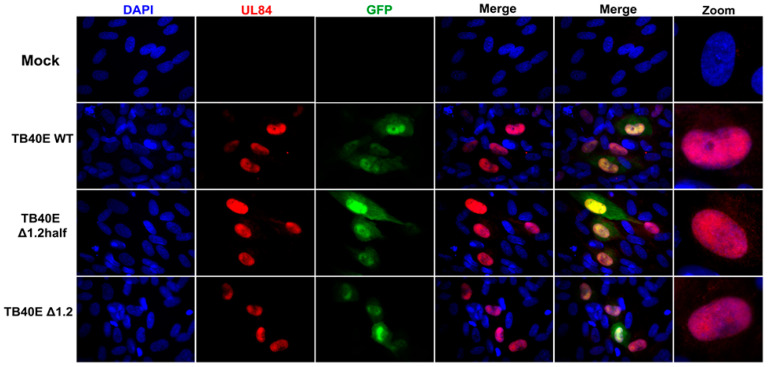
Viral protein localization is comparable between TB40E WT, TB40E ΔRNA1.2 half, and TB40E ΔRNA1.2. HF cells were infected with TB40E WT, TB40E ΔRNA1.2 half, and TB40E ΔRNA1.2 (MOI = 1). After 72 hpi, mock and infected cells were fixed and permeabilized. Cells were labeled with antibodies to detect viral proteins UL44, IE2, UL57, and UL84 (Red). Nuclei were stained with DAPI (blue). GFP expression can be detected in infected cells (green). Cells were mounted and imaged at 63x. Zoom panels are indicated on the right to show viral protein localization patterns.

As protein production did not seem to be altered in the absence of RNA1.2, we next assessed the localization of key viral proteins by immunofluorescent imaging (IFA) (Figure 3). HF cells were infected with TB40E WT, ΔRNA1.2 half, and ΔRNA1.2 for 72 h and then IFA was performed using antibodies specific to IE2, UL44, UL57, and UL84. IE2 expression is distributed throughout the host cell nucleus among all infections, similar to what has been previously reported. UL44 is known to concentrate with viral replication compartments and was found in these areas in WT and the RNA1.2 mutants. The viral encoded single-stranded binding protein, UL57, showed punctate formations and concentrated within the viral replication compartments. Lastly, the multifunctional protein UL84 was detected throughout the host nucleus for all samples. In summary, there were no defects or apparent changes in the localization of viral proteins in ΔRNA1.2 half and ΔRNA1.2 mutants compared to WT.

### 3.3. RNA1.2 Is Not Required for Productive Lytic Replication in Cell Culture

**Figure 4:**
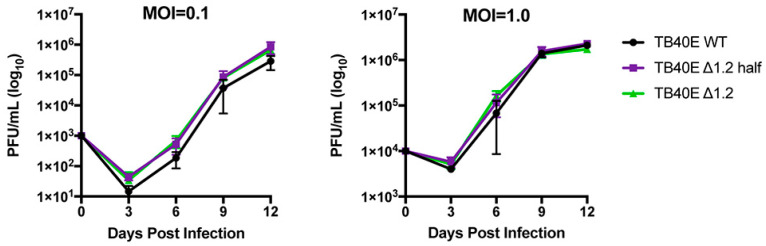
Growth curve analyses of TB40E WT, TB40E ΔRNA1.2 half, and TB40E ΔRNA1.2. HF cells were infected with TB40E WT, TB40E ΔRNA1.2 half, and TB40E ΔRNA1.2 at MOI = 0.1 and MOI = 1.0. Supernatants from infected cells were harvested every three days for up to 12 dpi. Infectious supernatant was titrated onto HF cells, and PFU/mL was calculated and plotted (N = 3).

Since gene expression and protein production were not altered in the ΔRNA1.2 mutants, growth curve analyses were performed to measure virion production at MOI = 0.1 and MOI = 1 in TB40E WT, ΔRNA1.2 half, and ΔRNA1.2 (Figure 4). The virus was collected every three days post-infection up to 12 dpi and titrated using plaque assays on HF cells. The number of plaque-forming units (pfu) was calculated per mL (PFU/mL) using three biological replicates and plotted on a logarithmic y-axis (log10). The growth curves showed no significant differences between TB40E WT, ΔRNA1.2 half, and ΔRNA1.2 at both tested MOIs. These data support the hypothesis that RNA1.2 is not required to produce infectious viruses during lytic replication in cell culture.

### 3.4. Capturing RNA1.2–Protein Complexes Using In Vitro Transcribed, Biotinylated RNA1.2

**Figure 5:**
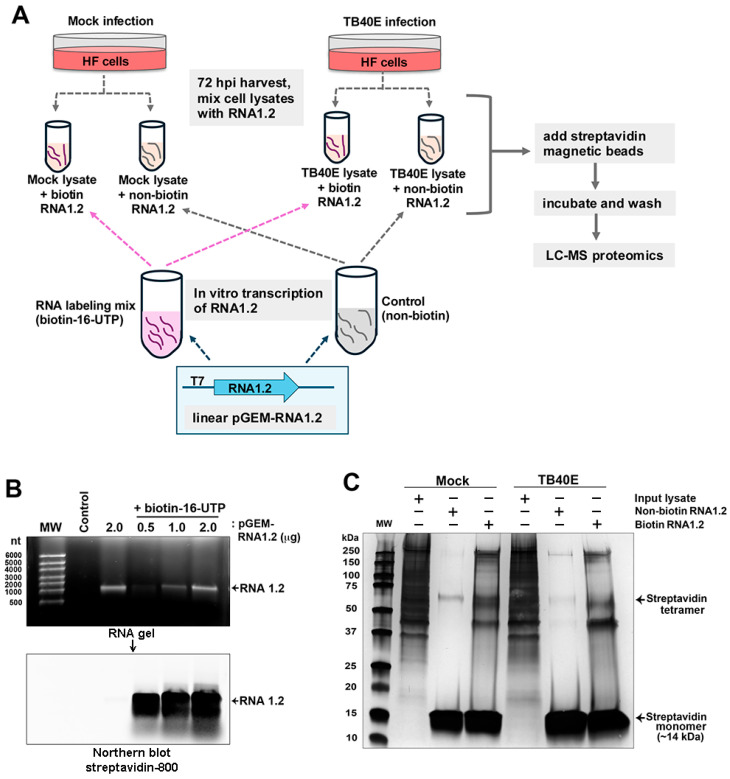
Biotinylated RNA1.2 interacts with the protein in mock and TB40E-infected cell lysate. (**A**) Diagram depicting the workflow for proteomics. Cells were mock-infected or infected with TB40E for 72 h before lysate was harvested and mixed with in vitro transcribed RNA1.2. (**B**) RNA1.2 was in vitro transcribed using linearized pGEM-RNA1.2 plasmid and an RNA labeling mix containing biotin-16-UTP or non-biotinylated control. The RNA transcript was isolated from the reaction mixture, and the size and quality of the RNA were assessed by gel electrophoresis (upper panel) and modified Northern blot using fluorescent labeled streptavidin (lower panel). The control sample contains no plasmid template. The Northern blot confirmed biotinylation of RNA1.2 transcripts. (**C**) Purified RNA1.2 was incubated with mock or TB40E-infected lysate, and the RNA–protein complexes were captured using magnetic streptavidin beads followed by extensive washing. Proteins interacting with RNA1.2 were eluted and resolved by SDS-PAGE followed by silver-staining to detect proteins.

Although our data in TB40E and other similar RNA1.2 mutants in strain Merlin have demonstrated that RNA1.2 is not required for viral replication in permissive fibroblasts in culture, it does not mean this transcript is truly dispensable during in vivo infections. To further investigate the potential functional role of RNA1.2 during infection, we sought to identify viral and cellular protein binding partners that interact with RNA1.2. Our approach was to use a biotinylated RNA1.2 as bait, incubated in mock and infected cell lysate, and streptavidin beads to capture the RNA–protein complexes (Figure 5A).

To generate biotinylated RNA, the RNA1.2 gene was cloned into the pGEM 7Zf(-) plasmid and used as a template for in vitro transcription with the T7 promoter. To incorporate biotin during the transcription of RNA1.2 from the template, a nucleotide triphosphate (NTP) labeling mix containing a fraction of the uridine-5′-triphosphate (UTP) available for RNA synthesis with the biotin modification (biotin-16-UTP) was used. By including a mix of native and biotin-modified UTP molecules, the structure of the RNA1.2 molecule is expected to be minimally affected. The synthesis of biotinylated RNA1.2 was confirmed by gel electrophoresis, and streptavidin was labeled for Northern blot analysis (Figure 5B). In the top panel, the gel electrophoresis image shows in vitro transcribed non-biotinylated RNA1.2 (2 µg) and biotinylated RNA1.2 (0.5–2 µg). The size of in vitro transcribed RNA1.2 is approximately 1200 nucleotides (nts) in length. A modified Northern blot was performed by incubating the electrophoresed samples with streptavidin-800 to visualize the biotinylated RNA, as shown in the bottom panel, to confirm the incorporation of biotin-16-UTP, and it was also noted that there was very minimal degradation of the RNA.

### 3.5. Identifying Viral and Cellular Proteins Associated with RNA1.2

After verifying the biotinylation of RNA1.2, we moved forward with isolating the protein–RNA complexes. The biotinylated RNA1.2 and control non-biotinylated molecules were incubated with mock- and TB40E-infected cell lysate (72 hpi). Protein–RNA complexes were isolated by streptavidin magnetic bead capture, followed by extensive washing. After the final wash, the beads were resuspended in Laemmli buffer and boiled, and the enriched proteins were resolved by SDS-PAGE. Silver staining of the gel showed substantial enrichment of proteins in samples incubated with biotinylated RNA1.2 versus non-biotinylated RNA1.2 (Figure 5C). There was protein detected in both the mock- and TB40E-infected cells, suggesting that many cellular proteins, regardless of the infection state, interact with RNA1.2.

**Figure 6:**
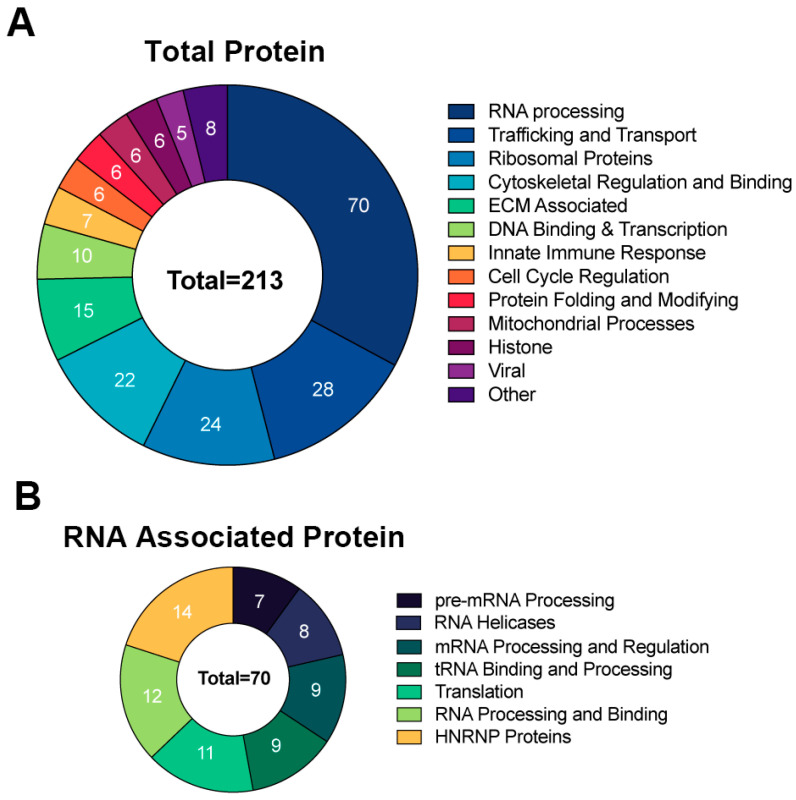
Comprehensive analysis of proteins associated with RNA1.2 during an HCMV infection. (**A**) Total proteins identified in proteomic analysis from the biotinylated RNA1.2 pull-down of associating proteins (N = 2). Protein hits were included if the enrichment ratio of biotinylated RNA1.2 versus non-biotinylated RNA1.2 was >3. (**B**) Categorization of proteins associated with RNA processing identified in the proteomic analysis described above.

To identify the proteins that interact with RNA1.2, the method above was used to isolate protein–RNA complexes and followed by liquid chromatography-mass spectrometry (LC-MS) and protein identification at the Mitch Hitchcock Nevada Proteomics Center, University of Nevada, Reno, according to their standard protocol. For analysis, proteins were considered potential RNA1.2 binding partners if the total peptide counts from biotinylated RNA compared to non-biotinylated RNA had an enrichment greater than three-fold. Averages of peptide counts were taken from two independent LC-MS experiments. The majority of proteins identified as interacting with RNA1.2 were cellular proteins (Figure 6). A pie chart representing the identified proteins categorized by function and type is shown in Figure 6A. Since cellular RNA binding proteins were most enriched, a separate pie chart is displayed to further categorize them into functional groups (Figure 6B).

**Figure 7:**
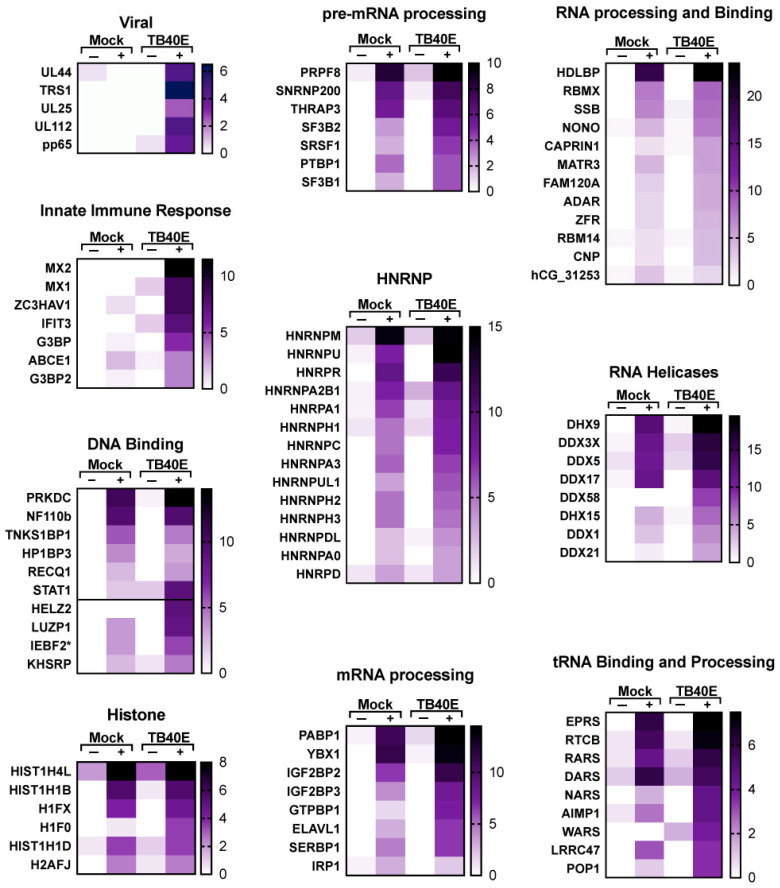
Enrichment of viral proteins and DNA/RNA binding proteins associated with RNA1.2 in mock- and TB40E-infected cells. Heat maps were used to compare the enrichment of RNA1.2-associated proteins from proteomic analysis of mock- and TB40E-infected cells at 72 dpi. Negative control samples “−” were incubated with non-biotinylated RNA1.2, and “+” samples were incubated with biotinylated RNA1.2 (N = 2). An asterisk indicates a protein variant with no gene name.

**Figure 8:**
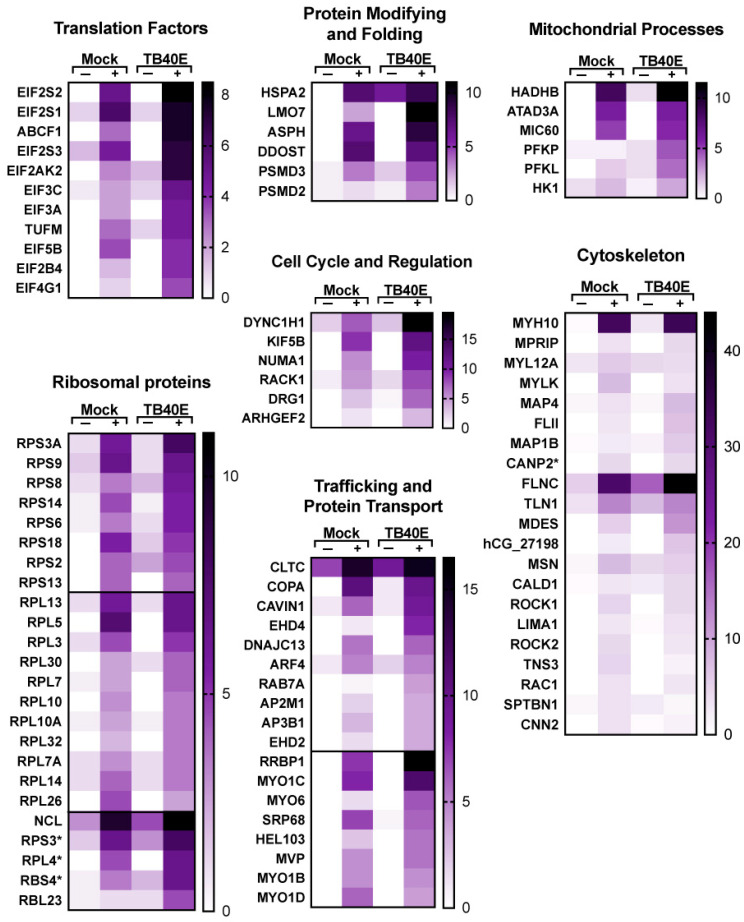
Enrichment of proteins involved in translation, modification and transport, and other cellular processes that interact with RNA1.2 in mock- and TB40E-infected cells. Heat maps were used to compare the enrichment of RNA1.2-associated proteins from proteomic analysis of mock- and TB40E-infected cells at 72 hpi. Negative control samples “−” were incubated with non-biotinylated RNA1.2, and “+” samples were incubated with biotinylated RNA1.2 (N = 2). Asterisks indicate protein variants with no gene name.

A more detailed representation of proteins enriched with RNA1.2 is represented as heat maps (Figure 7 and Figure 8). Of the total 213 proteins that showed more than three-fold enrichment from the biotinylated RNA1.2 compared to the non-biotinylated control, only five viral proteins were considered to be significantly enriched (Figure 7). Interestingly, most of the detected viral proteins are responsible for antagonizing the immune response and play a role in cell cycle control. Besides its classical role as a processivity factor, UL44 is reported to suppress transcription of antiviral genes, TRS1 inhibits autophagy, UL25 interferes with interferon-induced defense mechanisms, and tegument protein pp65 is involved in both innate and adaptive immune responses [34,35,36,37].

Many cellular RNA binding proteins were identified, including heterogeneous nuclear ribonucleoproteins (hnRNPs), RNA helicases, and RNA processing, and while it is likely that many of these are non-specific RNA–protein interactions, there is also a potential for RNA1.2 to regulate their activity during infection. A subset of cellular proteins that are particularly interesting are those associated with the innate immune response, mitochondrial processes, and cell cycle and regulation. Taken together, these findings indicate that RNA1.2 could modulate cellular functions, and this supports previous reports about the function of RNA1.2 with respect to regulating innate immune response [12].

**Figure 9:**
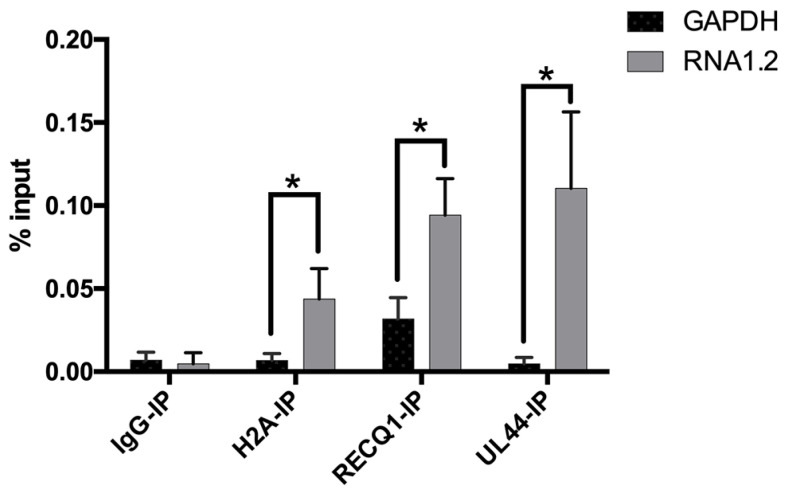
Detection of RNA1.2 interacting with viral and cellular proteins. HF cells were infected with TB40E. After 72 hpi, cell lysate was fixed and sheared. An RNA-IP was performed using antibodies to detect IgG, H2A, RECQ1, and UL44. RNA was isolated, and a qPCR analysis was performed to detect the RNA bound to protein for the IPs. In the qPCR analysis, the primers and probes used to detect transcripts were RNA1.2 and GAPDH. Percent input was calculated and graphed (N = 3). Error bars indicate the standard deviation from the mean. Statistical analysis was performed using Student’s *t*-test with multiple comparisons *, *p* < 0.05.

To further test some of the proteins identified in the LC-MS results, RNA immunoprecipitations (RNA-IP) were performed, and the enrichment of RNA1.2 was measured by qPCR. For this assay, lysate of TB40E infected cells (72 hpi) was incubated with antibodies specific to either H2A, RECQ1, UL44, or IgG control, and complexes were immunoprecipitated using protein G magnetic beads. RNA interacting with these proteins was isolated, and qPCR was performed to measure RNA1.2 and GAPDH transcripts (Figure 9). The percent input (% input) and standard deviation for RNA1.2 and GAPDH were calculated from three replicates for each RNA-IP. H2A, RECQ1, and UL44 showed significant enrichment of RNA1.2 compared to GAPDH, which validates the results from the RNA1.2 proteomic analysis.

## 4. Discussion

lncRNAs have proven to be key regulatory elements in viral and cellular biological processes. Investigation of these molecules has prompted a revolution in disease treatments and RNA-based therapeutics. As more studies emerge, these lncRNAs are shown to be significant in many diseases, such as neurodegenerative disease, cardiovascular disease, and cancers [38]. Next-generation treatments using therapies to either modulate lncRNA expression or to functionalize these non-coding transcripts for controlling DNA and protein expression are emerging [39,40,41]. Interestingly, in herpesvirus infections, lncRNAs are the most abundant transcripts produced [11,42,43]. This raises the question of whether viral lncRNAs are potential therapeutic targets for viral disease treatment.

For HCMV, the characterization of RNA1.2, RNA2.7, RNA4.9, and RNA5.0 has been at the center of research as the importance of lncRNAs has emerged. Our study determined that there were no viral defects of TB40E RNA1.2 mutants during a lytic infection by investigating the expression of viral genes and proteins and, ultimately, infectious virus production. We evaluated changes in viral transcript production between WT and RNA1.2 mutants. We analyzed protein production and localization of viral IE, E, and L proteins. There were no significant defects in the production of any viral products. The viral growth curve analyses showed that viral infectious output was unaltered without RNA1.2.

Prior to our current study, there was a report using RNA1.2 mutants made in the HCMV Merlin strain with deletions to part of the RNA1.2 locus or the TATA promoter element [12]. Their data determined that the RNA1.2 mutants had minimal changes in viral transcript expression by performing transcriptome sequencing, and they also found no change in viral replication or virion production and release in fibroblast cell culture. Interestingly, a more recent study using RNA1.2 deletion mutants in strain Toledo found RNA1.2 required for lytic replication and noted a two- to ten-fold reduction in the amount of virus produced, consistent with the lower relative amount of viral proteins [28]. In our study, we focused on characterizing an RNA1.2 mutant with either the whole RNA1.2 locus removed or the 3′-half. Future studies using deletions within the 5′-half or small nucleic acid changes within RNA1.2 will help define specific functional domains of RNA1.2.

Since protein binding partners for lncRNAs can often play a role in their function, we sought to identify protein binding partners of RNA1.2. We used biotinylated RNA1.2 as bait to capture proteins in mock-infected and infected cell lysate. This allowed us to identify any proteins and complexes associated with RNA1.2. It is interesting to note that the few viral proteins that we identified, UL44, TRS1, UL25, UL112, and pp65, were not the same viral proteins identified by Lee et al., who reported IRS1, UL35, and UL47 [28]. This could be due to the difference in the approach to isolating the RNA–protein complexes in which we used an RNA-IP method. The Lee et al. study used a modified RNA antisense purification coupled with mass spectrometry (RAP-MS) [28].

Prior studies performed by Lau B. et al. suggest that RNA1.2 plays a significant role in evading the host immune response [12]. Their results suggested that RNA1.2 modulates the host immune response by interfering with NF-κB-dependent cytokine and chemokine release. This was intriguing, since our proteomic studies revealed a subset of innate immune defense protein enriched with RNA1.2 in infected cells. Interacting with immune regulators would also explain why RNA1.2 is highly expressed but does not display a defect in cell culture replication. The most enriched immune proteins interacting with RNA1.2 detected were MX1 and MX2 (MXA and MXB). MX proteins are ubiquitous in vertebrates and have very broad antiviral activity. The expression of *mx* genes is stimulated by interferon signaling, which explains their increased expression during HCMV infection [44]. Their mechanism has been significantly described for RNA viruses and more recently for herpesviruses [44,45]. Studies have shown that upon infection in HSV-1 and KSHV herpesviruses, MXB can inhibit viral DNA uncoating and entry from the capsid [46]. Therefore, one potential role of RNA1.2 is to inhibit the function of these immune-response proteins by binding and physically inhibiting the mechanistic actions of MXA and MXB.

Another interesting subset of proteins we identified were those associated with mitochondrial processes. RNA2.7 has been previously reported to interact with GRIM19 of mitochondrial complex-1 to modulate cellular metabolism and mediate the host apoptotic pathway [18]. We did not specifically detect GRIM19 in our results, but we did find interaction with a protein associated with the mitochondrial trifunctional enzyme complex. HADHB, the b-subunit of this complex, was the most significantly enriched form of the identified mitochondrial proteins. This protein is involved in the β-oxidation pathway of fatty acids [47]. In support of our findings, a previous report identified manipulation of this pathway during HCMV infection to enhance viral infectivity [48]. It is known that during HCMV infection, the host mitochondria become morphologically and functionally altered [49]. Upon infection, mitochondria undergo a hyperactive state where there is increased fission, glycolysis, respiration, and production of stress markers. RNA1.2 likely has a function in manipulating mitochondria to augment infection.

Many other cellular proteins were identified in the proteomic analysis, but these binding interactions may not all have a purpose or function. It is likely that a multitude of these cellular proteins we detected broadly bind to RNA1.2 because it is an RNA molecule. Further studies examining the specific interactions of these cellular proteins binding with RNA1.2 will be integral for gaining insights into host–viral interactions. It is important to mention that this study only revealed the comprehensive discovery of protein-binding partners and does not discuss the potential of RNA1.2 binding to other RNA molecules or genomic DNA. Experiments identifying the association of RNA1.2 with the viral or cellular nucleic acids can reveal novel viral–host interactions. This further amplifies the complexity of lncRNAs during viral infection and emphasizes the importance of understanding their functions.

## Figures and Tables

**Table 1:** Sequences of gblock synthesis for viral BACmid recombineering.

Name	Type	Sequence
∆1.2 +KAN	gBlock	5′-AGATTTCATCAGGTTTATTTTGGCTGCTGCTAGTCTTTTGCTTCGGACACTCGCGTCCGGTTGGGCCG ATTTATTCAACAAAGCCACGTTGTGTCTCAAAATCTCTGATGTTACATTGCACAAGATAAAAATATATC ATCATGAACAATAAAACTGTCTGCTTACATAAACAGTAATACAAGGGGTGTTATGAGCCATATTCAAC GGGAAACGTCTTGCTCGAGGCCGCGATTAAATTCCAACATGGATGCTGATTTATATGGGTATAAATGG GCTCGCGATAATGTCGGGCAATCAGGTGCGACAATCTATCGATTGTATGGGAAGCCCGATGCGCCAG AGTTGTTTCTGAAACATGGCAAAGGTAGCGTTGCCAATGATGTTACAGATGAGATGGTCAGACTAAA CTGGCTGACGGAATTTATGCCTCTTCCGACCATCAAGCATTTTATCCGTACTCCTGATGATGCATGGTT ACTCACCACTGCGATCCCCGGGAAAACAGCATTCCAGGTATTAGAAGAATATCCTGATTCAGGTGAA AATATTGTTGATGCGCTGGCAGTGTTCCTGCGCCGGTTGCATTCGATTCCTGTTTGTAATTGTCCTTTTA ACAGCGATCGCGTATTTCGTCTCGCTCAGGCGCAATCACGAATGAATAACGGTTTGGTTGATGCGAG TGATTTTGATGACGAGCGTAATGGCTGGCCTGTTGAACAAGTCTGGAAAGAAATGCATAAGCTTTTG CCATTCTCACCGGATTCAGTCGTCACTCATGGTGATTTCTCACTTGATAACCTTATTTTTGACGAGGGG AAATTAATAGGTTGTATTGATGTTGGACGAGTCGGAATCGCAGACCGATACCAGGATCTTGCCATCCT ATGGAACTGCCTCGGTGAGTTTTCTCCTTCATTACAGAAACGGCTTTTTCAAAAATATGGTATTGATA ATCCTGATATGAATAAATTGCAGTTTCATTTGATGCTCGATGAGTTTTTCTAATCAGAATTGGTTAATT GGTTGTAACACTGGCATTACCCTGTTATCCCTAGATCGATGTACGGGCCAGATATACGCGGCTGCTG CTAGTCTTTTGCTTCGGACACTCGCGTCCGGTTGGGCATTGCCCACAGGAAGATGAGTC-3′
∆1.2 half+KAN	gBlock	5′-CAGACCACTGGGAGTTCAGTTAAAGATTTCATCAGGTTTATTTTGAGGGCGCGGTCATCTTTTACTC GATTTATTCAACAAAGCCACGTTGTGTCTCAAAATCTCTGATGTTACATTGCACAAGATAAAAATATA TCATCATGAACAATAAAACTGTCTGCTTACATAAACAGTAATACAAGGGGTGTTATGAGCCATATTCA ACGGGAAACGTCTTGCTCGAGGCCGCGATTAAATTCCAACATGGATGCTGATTTATATGGGTATAAAT GGGCTCGCGATAATGTCGGGCAATCAGGTGCGACAATCTATCGATTGTATGGGAAGCCCGATGCGCC AGAGTTGTTTCTGAAACATGGCAAAGGTAGCGTTGCCAATGATGTTACAGATGAGATGGTCAGACTA AACTGGCTGACGGAATTTATGCCTCTTCCGACCATCAAGCATTTTATCCGTACTCCTGATGATGCATG GTTACTCACCACTGCGATCCCCGGGAAAACAGCATTCCAGGTATTAGAAGAATATCCTGATTCAGGT GAAAATATTGTTGATGCGCTGGCAGTGTTCCTGCGCCGGTTGCATTCGATTCCTGTTTGTAATTGTCCT TTTAACAGCGATCGCGTATTTCGTCTCGCTCAGGCGCAATCACGAATGAATAACGGTTTGGTTGATG CGAGTGATTTTGATGACGAGCGTAATGGCTGGCCTGTTGAACAAGTCTGGAAAGAAATGCATAAGCT TTTGCCATTCTCACCGGATTCAGTCGTCACTCATGGTGATTTCTCACTTGATAACCTTATTTTTGACGAG GGGAAATTAATAGGTTGTATTGATGTTGGACGAGTCGGAATCGCAGACCGATACCAGGATCTTGCCA TCCTATGGAACTGCCTCGGTGAGTTTTCTCCTTCATTACAGAAACGGCTTTTTCAAAAATATGGTATT GATAATCCTGATATGAATAAATTGCAGTTTCATTTGATGCTCGATGAGTTTTTCTAATCAGAATTGGTT AATTGGTTGTAACACTGGCATTACCCTGTTATCCCTAGATCGATGTACGGGCCAGATATACGCGAAGA TTTCATCAGGTTTATTTTGAGGGCGCGGTCATCTTTTACTTTTCGGTTTTCTCATTGGCGGG-3′

**Table 2:** Primer and probe sequences for qPCR analysis.

Name	Sequence
	Primer 1: 5′-GCCGAGAGAATGCCAGTAAG-3′
RNA 1.2	Primer 2: 5′-CTGCTGTACGTGTGATGGTTAT
	Probe: /56-FAM/CGT ACT GTG/ZEN/TCT GCG ATG GTC GTC/3IABkFQ/
	Primer 1: 5′-GTGTGTGCTGGCCGATG-3′
RNA 4.9	Primer 2: 5′-GGGACGGTGATTGTGGAG-3′
	Probe: /56-FAM/ACC TCA ATT/ZEN/GTC GTC AGT ACG CCC/3IABkFQ/
	Primer 1: 5′-GCGAAGAAGTGCGAGGATAA-3′
Beta 2.7	Primer 2: 5′ CATCATCATCGGAGACCATCTT-3′
	Probe: /56-FAM/AAA TGG ATG/ZEN/ACT CCT TCG TGT CCA GG/3IABkFQ/
	Primer 1: 5′-GCGAAGAAGTGCGAGGATAA-3′
RNA 5.0	Primer 2: 5’-AACATCATCGGAGACCATCTT T-3′
	Probe: /56-FAM/AAA TGG ATG/ZEN/ACT CCT TCG TGT CCA GG/3IABkFQ/
	Primer 1: 5′-GAAAGAAGAGGACGAGGATGAC-3′
IE2 ex5	Primer 2: 5′-GTGCGGGAAAGAGAGAGAAG-3′
	Probe: /56-FAM/TCT AAC GAG/ZEN/GAT TCT GAC GTG CGC/3IABkFQ/
	Primer 1: 5′-GTAGTGGTTGGGCAGGATAAA-3′
UL54	Primer 2: 5′-TTGCGGCGTGTCATCTT-3′
	Probe: /56-FAM/TAT CTA CAC/ZEN/CTC GCT GCT GGA CGA/3IABkFQ/
	Primer 1: 5′-TGCGTAAAGTCGAAGAAGGG-3′
UL86	Primer 2: 5′-CGCACGGTGAACGAAATAAAG-3′
	Probe: /56-FAM/CAA GGT GGG/ZEN/CAA CAT CAC GCT CTA/3IABkFQ/
	Primer 1: 5′-TGACTACCCTACGTTCTCCTAC-3′
7sk	Primer 2: 5′-GTCAAGGGTATACGAGTAGCTG-3′
	Probe: /56-FAM/CCC TGC TAG/ZEN/AAC CTC CAA ACA AGC T/3IABkFQ/
	Primer 1: 5′-TGTAGTTGAGGTCAATGAAGGG-3′
GAPDH	Primer 2: 5′-ACATCGCTCAGACACCATG-3′
	Probe: /56-FAM/AAG GTC GGA/ZEN/GTC AAC GGA TTT GGT C/3IABkFQ/

**Table 3:** Sequences of gblock synthesis for plasmid recombination.

Name	Type	Sequence
pGEM-RNA1.2	g-Block	5′-CCCGACGTCGCATGCTCCTCTAGAAGTGTCCCATAAAAGCCGGGCGCTCCGGCGAGACCATGCCA TCCTCGCCTTCGGACGCCCCGCTCCTCTTCTCTCTCCTCTCCTCCCCGCTGCCGCGGCCACTGCCGCC GCCGCCCATACCATCGGCATGTCGGCCGACAAATCGCAGCTGTCGTCGTCGCCGCCGCAGCTGTAGC AGTTAACGTCGCCGGCCTTCAGGAGGAGATGGCGCTCTGCGTCGTCTCTTCGTCCCGCCTCCCTCTGT GGTCGTGGGTGGTGCGAGAGTACACGATGGGTGGCTCTCGTCTCGGGGGACCACAGGGGGAGGGGG GTAATTTATTATTCGTATTACTGTAATTTTGTATCGCTTAATTTGTTTAGAGCCGCACGCTTGACAACGC CTTGTATAGCCTTATTTATCCCGATGACTTTTTTCTCCGTACAAGAAATGGACGTCACTTGAGCAGAC ACAGTTTCATCGACCACGACAGTCTCATGATCTGACTACCTCTGACCCGCCAATGAGAAAACCGAA AAGTAAAAGATGACCGCGCCCTCGGAGTCCTTTTTTCCTTTTCAATCATGAAAGCAAGAGGCAGC CGAGAGAATGCCAGTAAGAGACGACCATCGCAGACACAGTACGATACTCATCTTAGAACGAACCA GCGAATAACCATCACACGTACAGCAGAATCTCATGAACTAGTCAACCAACGTCATAAAATCTTCA CACAATCGTTTTTGCGAACTTTTAGGAACCAGCAAGTCAACAAAAGACTAACAAAGAAAAACCA TCTTGGAATTAAAAAAAGTAGCATCGTTACCTTATGAACCAGCAGCATTCAGTATATACACCAGAT ATAATATATTTATTAATGTATCCTCTCTTTCTCCTGATGTAATTTTGTTTTTGTAAATTCAATTGTTGA AAGTCTCTCCCTGGGGGAATTGCATATCTTATTGATGAAGAAGAAATCCCTGCCATATGTGTTGTC AAACTATCATTATTTCTCTATATGGGTATTTTTTTTCTAAGAAGCAAAAGACTAGCAGCAGCCA AAATAAACCTGATGAAATCTTTAACTGCTCGAGGAATTCGGTACCCCGGGT-3′

## Data Availability

All data supporting the findings of this study are provided within the manuscript and its Supplementary Materials.

## Supplementary Materials

The following supporting information can be downloaded at: https://www.mdpi.com/article/10.3390/v17020149/s1, Supplemental Table S1: RNA1.2 proteomics is submitted with the manuscript.

## Abbreviations

The following abbreviations are used in this manuscript:

HCMV	Human Cytomegalovirus
lncRNA	Long Non-Coding RNA
BAC	Bacterial Artificial Chromosome
NF-κB	Nuclear Factor Kappa-Light-Chain-Enhancer of Activated B Cells
IL-6	Interleukin 6
DRS	Direct RNA Sequencing
ORFs	Open Reading Frames
GCV	Ganciclovir
VGV	Valganciclovir
MBV	Maribavir
IE	Immediate-Early
E	Early
L	Late
MIEP	Major Immediate-Early Promoter
PCR	Polymerase Chain Reaction
LC-MS	Liquid Chromatography-Mass Spectrometry
MOI	Multiplicity of Infection
ATP	Adenosine Triphosphate
DAPI	4′,6-Diamidino-2-Phenylindole
TE	Tris-EDTA Buffer
hpi	Hours Post-Infection
PBS	Phosphate Buffered Saline
RIP	RNA Immunoprecipitation
TBST	Tris Buffered Saline with Tween-20
FDR	False Discovery Rate
